# Palliative Care Clinician Perspectives on Person-Centered End-of-Life Communication for Racially and Culturally Minoritized Persons with Cancer

**DOI:** 10.3390/cancers15164076

**Published:** 2023-08-12

**Authors:** William E. Rosa, Meghan McDarby, Haley Buller, Betty R. Ferrell

**Affiliations:** 1 Department of Psychiatry and Behavioral Sciences, Memorial Sloan Kettering Cancer Center, New York, NY 10017, USA; mcdarbym@mskcc.org; 2 City of Hope, Duarte, CA 91010, USA; hbuller@coh.org (H.B.); bferrell@coh.org (B.R.F.)

**Keywords:** antiracism, communication, end of life, pain, palliative care, racism

## Abstract

**Simple Summary:**

Racially and culturally minoritized persons with serious illness receive subpar and potentially harmful care at end of life. Despite health equity initiatives, little is known about how palliative care clinicians perceive and engage in serious illness communication with persons from racially and culturally minoritized groups. The aim of this study was to explore how 152 nurses, social workers, and chaplains would prioritize communication with a Black, Native American woman with a history of experiencing structural racism and report of severe pain at end of life. Clinicians’ responses were thematically analyzed and reflected the following themes: person-centered, authentic, and culturally-sensitive care; pain control; approaches to building trust and connection; and understanding the communication challenges related to racial differences. Findings support the need for additional interventions that address clinicians’ unconscious biases, better integrate culturally inclusive communication in practice, and improve the quality of end-of-life care for persons from minoritized groups.

**Abstract:**

The aim of this study was to examine interdisciplinary clinicians’ perceptions of priorities in serious illness communication and shared decision-making with racially and culturally minoritized persons at end of life. Clinicians (*N* = 152) read a detailed case study about a patient self-identifying as Black and American Indian who describes mistrust of the healthcare system. Participants then responded to three open-ended questions about communication strategies and approaches they would employ in providing care. We conducted a thematic analysis of participants’ responses to questions using an iterative, inductive approach. Interdisciplinary clinicians from nursing (48%), social work (36%), and chaplaincy (16%), responded to the study survey. A total of four themes emerged: (1) person-centered, authentic, and culturally-sensitive care; (2) pain control; (3) approaches to build trust and connection; and (4) understanding communication challenges related to racial differences. Significant efforts have been made to train clinicians in culturally inclusive communication, yet we know little about how clinicians approach “real world” scenarios during which patients from structurally minoritized groups describe care concerns. We outline implications for identifying unconscious bias, informing educational interventions to support culturally inclusive communication, and improving the quality of end-of-life care for patients with cancer from minoritized groups.

## 1. Introduction

Palliative care is a person-centered, interdisciplinary approach for patients with serious illness and their families [1] that is correlated with increased quality of life, decreased health costs, and fewer hospital admissions [2,3]. “Families” hereafter is used to describe biological or chosen family, as well as other informal caregivers or surrogate decision-makers for patients with serious illness [4,5]. The provision of palliative care can be integrated into treatment plans in conjunction with disease-focused interventions to address biopsychosocial, cultural, and spiritual needs starting at time of serious illness diagnosis, continuing through end of life (EOL) and, ideally, into bereavement. 

Palliative care provision and outcomes for racially and culturally minoritized persons (e.g., Black and Native American people) with serious illness are substandard when compared to non-minoritized persons. For instance, care inequities for Black and Native American patients and their caregivers exist across the palliative care spectrum, including but not limited to poorer symptom management, disparate medically indicated treatments for pain (e.g., equitable opioid prescribing and monitoring practices) [6,7,8,9,10,11], discriminatory and biased clinician behaviors and communication [12,13,14], poor integration of patients’ spiritual and cultural values into care delivery [15,16], less high-quality evidence and documentation of advance care planning [17,18,19], and significant mistrust of the health system [20,21] that impacts patients’ decision making and relationships with clinicians. Such disparities lead to poorer clinical outcomes for patients (e.g., pain, goal discordant care) and caregivers (e.g., bereavement). Many palliative care researchers and professional organizations have committed to dismantling structural barriers that prevent equitable access, calling for culturally-responsive and community-based social justice methods and adoption of antiracist principles in all domains of practice [22,23,24,25].

Communication capacity building and training is one potential avenue for reducing disparities. Empathic communication is the foundation of high-quality palliative care, helping clinicians to effectively elicit patient and family goals, values, preferences, and needs [1,26,27]. For example, bereaved family members of Black decedents who reported better communication quality with clinicians showed lower decisional conflict during EOL care [28], which may have implications for decreasing symptoms of prolonged grief disorder. Approaches to improving communication for Black patients have included a community-based serious illness communication guide adaptation [29], partnership with local faith communities to strengthen psychosocial and spiritual support during decision-making [30,31], development of race-conscious serious illness communication strategies [32], communication-priming goals of care discussions for hospitalized patients, [33] and increased unconscious bias and cultural humility training [24]. 

Evidence-based communication strategies are key to optimizing patient and family outcomes in the oncology and palliative care contexts [34,35,36,37,38]. Despite well-documented pain and health service utilization disparities, there is scant data regarding interdisciplinary clinicians’ communication knowledge and priorities when caring for patients from racially and culturally minoritized groups at EOL. This study aimed to fill this critical gap. 

## 2. Materials and Methods

### 2.1. Participants and Procedure

Participants in the current study were interdisciplinary cancer clinicians (*N* = 152) from across the United States attending the National Cancer Institute (NCI)-funded Interdisciplinary Communication Curriculum (ICC) Project. The ICC is a 3-day, in-person communication skills training program for interdisciplinary oncology clinicians, including nurses, social workers, and chaplains. ICC faculty are senior leaders from nursing, social work, and chaplaincy, and they all hold expertise in communication skills. The main objectives of the ICC course are supporting participants in their ability to (a) understand key domains of quality palliative care, (b) understand and perform effective clinical communication skills, and (c) establish goals to effectively implement communication skills in their daily practice. Primary learning activities in the course include conversation guides, role-play activities, and case studies. The format of the course is dynamic and includes didactics, discussion sections, and small-group activities.

The National Consensus Project for Quality Palliative Care clinical practice guidelines [1] were used to build a foundation for the ICC curriculum and training. Because these guidelines specifically emphasize cultural aspects of care, approximately two hours of trainees’ participation is directly related to cultural care. Participation related to cultural care includes a lecture about culturally inclusive care, a 1 h lab session to practice culturally sensitive communication skills, and a small-group case study review. Cultural considerations are also addressed in each of the other modules—for example, via discussion of cultural factors influencing pain and symptoms in the ICC module on symptom management. Participants from two ICC courses were invited to complete a short online survey immediately following the 3-day course (*n* = 94) or at 12 months post course (*n* = 58). Study recruitment and procedures were deemed exempt by the City of Hope IRB given that all data were collected from course participants only (assurance number 00000692).

### 2.2. Measures

Participants provided written responses to three open-ended questions pertaining to a cultural diversity-focused case study about an older adult (71 years old) woman self-identifying as Black and American Indian presenting for care associated with end-stage ovarian cancer. Based on key literature and the ICC course content, the composite case was developed by authors WER and BRF (experts in palliative and end-of-life care communication) and then reviewed by MM and HB. The patient in the case study describes reluctance to seek medical care due to lack of trust in the health care system and her experiences of racism from medical providers. The complete case summary and case questions are presented in Table 1.

### 2.3. Data Analysis

Two authors (BF, HB) conducted a thematic analysis using an iterative, inductive approach. Coding was guided by the overarching research question, “What do interdisciplinary clinicians cite as key components and priorities of serious illness communication with patients from minoritized racial and cultural groups?” Researchers BRF and HB coded participants’ responses to each of the three questions separately, generating unique codes for every question, which were then collapsed iteratively into unique themes for every question. The entire research team (all study authors) then organized themes for each question into overarching themes and identified key properties of each theme across all three questions. 

During early coding, BRF and a research coordinator met together and used a consensus approach to apply codes to individual participant responses. The coding team repeated this process for all participant responses to question 1 until an understanding of how to apply codes had been reached. A single member of the coding team independently coded the remaining participant responses, then met with the other coder to review codes. Each coder created memos about key themes and observations emerging from participant responses.

After initial coding, codes were collapsed within each question, then into themes. The entire group of authors discussed key findings as a team and identified prominent themes within each question, then examined commonalities in themes across questions. Throughout discussions between the coding team and immersion in participant responses, we identified four themes and collapsed codes into core properties representing each theme. Thematic saturation was reached and was established based on criteria of forcefulness, repetition, and recurrence [39]. 

## 3. Results

### 3.1. Participant Characteristics 

Participants were predominantly female (86%) and identified as White (65%). Most participants identified as nurses (48%), followed by social workers (36%) and chaplains (16%). See Table 2 for additional demographic information.

### 3.2. Qualitative Themes

We identified four themes: (1) person-centered, authentic, and culturally-sensitive care; (2) pain control; (3) approaches to build trust and connection; and (4) understanding communication challenges related to racial differences. Participants’ responses characterize each theme, as well as specific properties of each theme, and provide additional depictions of clinicians’ priorities in communicating with patients from racially and culturally minoritized groups and their caregivers (Table 3). Participant responses captured in themes highlighted that person-centered care is a goal but is often not achieved for patients from racially and ethnically minoritized groups.

#### 3.2.1. Theme 1: Person Centered, Authentic, and Culturally-Sensitive Care

Participants described the importance of delivering person-centered care and communication to the patient, Diane, described in the case study. Responses emphasized the specific value of offering authentic care, such that it would be clear to the patient that the clinician was coming from a place of genuineness and compassion when providing care. Participants went as far as to say that they would explicitly state their desire to help the patient (e.g., “We want to help you”.) to make their intentions and approach very clear to the patient. Notably, participants also emphasized the importance of validating the patient and her experiences, focusing on processes including acknowledging her pain and history, normalizing demonstrating humility about her feedback about her care experiences. 

#### 3.2.2. Theme 2: Pain Control

Participants often reported that a key component of their communication with the patient would involve a focus on pain control and pain management. Their responses reflected participants’ attentiveness to key parts of the case vignette, including the patient’s history with pain control and the patient’s beliefs and preferences about pain control and treatment. Some participants described the importance of assessing the patient’s perspective about pain medication and eliciting information about experiences that may have informed their perspectives. Others pointed to the relevance of asking the patient to explain their fears about pain and pain management. In terms of pain control, many participants also referenced the need to provide education, information, and resources to patients about pain. Some responses also highlighted the role of the clinician to instill self-efficacy about pain management post-discharge or throughout the illness trajectory.

#### 3.2.3. Theme 3: Approaches to Build Trust and Connection

Implicit in participants’ responses was an understanding that trust and connection would be critical to build a positive therapeutic relationship with the patient, as well as with patients from other racially and culturally minoritized groups. Participants explicitly referenced that they would offer assurance to demonstrate their commitment to the patient’s care, and that by demonstrating this commitment, they could convey trust and connection. Broadly, participants also spoke of the importance of inviting the patient’s perspectives about care and treatment by asking questions about their needs, preferences, and previous experiences. Responses indicated that participants could use the patient perspective to build trust and connection by asking clarifying questions and maintaining a focus on the patient’s desires for care and support. Participant responses also emphasized the importance of using their language and communication style to convey safety, compassion, and empathy to their patients. Perspectives emphasized creating a “safe space,” “affirming the patient’s identity as an expert,” and allowing time for patient questions, plus responding to those questions openly and honestly.

#### 3.2.4. Theme 4: Understanding Communication Challenges Related to Racial Differences

Most participants pointed to several communication challenges inherent to working with racially and culturally minoritized patients. Participants described three convergent challenges related to communicating with patients from racially and culturally minoritized backgrounds: challenges affecting patients, challenges affecting clinicians, and challenges affecting the patient–clinician dynamic. 

Primarily, the need for clinicians to acknowledge the scope of their own biases and judgments, as well as biases that patients may hold toward medical care based on previous experiences, was something noted by participants to be of utmost importance. Participants spoke of the importance of clinicians addressing their own biases and judgments and holding in awareness beliefs that patients may bring into encounters. Relatedly, participants noted the importance of clinicians having training in cultural competence and working with patients from different racial and ethnic backgrounds. Their comments included concern when clinicians did not have experience—either personal or professional—working with individuals from different backgrounds than their own. Clinicians in the study also described their own fears about saying something offensive to a patient or using insensitive language. Some participants noted that by making a language or communication blunder, they could potentially threaten the patient–clinician relationship. 

On the patient side, participants reflected a belief that it is important to acknowledge, understand, and validate patient experiences of mistrust and uncertainty with clinicians. Participants were aware that feelings of mistrust held by patients could significantly affect their communication and care. Participants also referenced challenges to the patient–clinician relationship affecting communication, including the issue of patient and clinician identity non concordance. Some participants described their perception that patients may not feel comfortable sharing information with them if they were a different race, or that patients might find language barriers complicated. 

## 4. Discussion

This study uses a case-based approach to explore the communication knowledge and priorities of palliative nurses, social workers, and chaplains caring for a Black and Native American woman at EOL with severe cancer pain and a history of racism and health system mistrust. Participant responses pointed to significant clinician discomfort in dismantling racism through interpersonal communication while providing dignified care for patients of color. Conversely, participants were self-reflective in their own limitations when providing culturally sensitive care, acknowledging the need for additional training and personal and professional development. Four themes were apparent throughout the qualitative responses, including person-centered, authentic, and culturally-sensitive care; pain control; approaches to build trust and connection; and understanding communication challenges related to racial differences. Findings can inform future communication training content for interdisciplinary palliative care clinicians, health teams, and systems that intend to promote culturally safe care provision and create environments that foster clinician self-reflection and assessment of bias. 

Empathic communication is central to person-centered, authentic, and culturally-sensitive palliative care (theme 1). The strategies, skills, and process tasks that characterize empathic communication have been shown to mitigate stigma and perceived bias for several populations (e.g., geriatric [35], lung cancer [36]), while moderating quality of life [40] and assisting clinicians to organize information and more confidently discuss complex clinical situations [37,41]. Although the need for empathic communication relates to clinical practice for all populations writ large, we provided a hypothetical composite case that specifically asked clinicians to consider potentially difficult and sensitive topics such as their communication responses to a patient-reported history of racism and discrimination. Empathic communication knowledge and priorities may become increasingly complex for clinicians when the hypothetical patient’s mistrust due to prior mistreatment (e.g., hesitant to take opioids and lose autonomy) is at odds with their worst fear (e.g., dying in pain). Researchers have found that Black patients with serious illness confront high rates of microaggressions and often have their self-knowledge and lived experiences dismissed by clinicians [14]. Our results suggest that by eliciting the patient’s story, validating their experiences, and responding authentically—while addressing their concerns and worries about the racism they encounter—empathic communication that is person-centered, authentic, and culturally sensitive can be a crucial part of antiracist palliative care practice. 

The second theme identified in this current study was pain control. Many people of color—including persons from minoritized racial and cultural backgrounds—experience well-documented cancer pain disparities [6,10,42]. Clinician participants frequently suggested a need to better understand patients’ perspectives of pain and pain control, and to provide tailored education to ensure pain management is aligned with evidence-based guidelines while honoring personal values. As a result, future research and clinician communication skills training endeavors should prioritize the delivery of information about the use of empathic communication skills to validate, normalize, and demonstrate understanding for minoritized patients’ experiences of marginalization in health care systems. Furthermore, experiential learning exercises for clinicians participating in communication skills training programs should embed racially- and culturally-specific needs into standardized patient assessment activities, including addressing mistrust and other barriers to communication for minoritized patient groups. These initiatives may address challenges to both person-centered care and pain control. 

In the current study, the patient described in the case example reported a history of mistreatment within and mistrust of the health system informed by experiences of racism. Though some participants acknowledged why these factors could make care more challenging and difficult for the patient, many participants conveyed discomfort and uncertainty with how to address and engage the patient in this conversation about prior mistreatment. Evidence-based navigation of such conversations should be a required component of clinical training to support high-quality palliative care, which includes attention to the social determinants of health, cultural values, and identity [1]. The study participants—nurses, social workers, and chaplains—all have diverse and unique roles in the health system that can address various aspects of building trust, connection, and rapport to support patients who feel physically or psychologically unsafe and forging clinical cultures that demonstrate a commitment to inclusion (theme 3: approaches to building trust and connection). In addition, participant responses support the need for social history taking as a vital skill for all clinicians so they may better understand patient behaviors, beliefs, values, and the lived experiences that inform how they perceive and receive health services (theme 4: understanding communication challenges related to racial differences). 

A number of trainings, technology advancements, and collaborations with colleagues (e.g., interpreters) have stressed the need for improved cross-cultural communication among clinicians [41,43,44,45,46,47]. For instance, the culturally competent communication model (CCM) provides competencies foundational to inclusive communication for diverse populations, including: (1) epidemiological knowledge and treatment effects across groups; (2) awareness of cultural influences on persons’ behavior and thinking; (3) awareness of persons’ social context; (4) awareness of one’s own prejudices and stereotypes; (5) ability to communicate in a way that is understandable and accessible to the patient; and (6) ability to adapt to new circumstances with flexibility and creativity [48]. When considering cancer communication specifically, it is essential to consider and address the underrepresentation of minoritized racial and ethnic groups in communication science, [49] as well as unique needs at individual and community levels (e.g., respecting traditional knowledge) [50]. Our study provides crucial interdisciplinary clinician perspectives to inform future cultural competency and cultural humility communication training and capacity building to promote person-centered and antiracist palliative care provision.

Research implications include the need to better develop stakeholder and community-informed serious illness communication guides and tools that actively address all levels of racism, personal and social histories of structural discrimination, and recognize minoritized racial and cultural identity as a social determinant of health. Community-based participatory research approaches have proven useful in ensuring patient, family, and community priorities and values into palliative care interventions for people of color [23,51,52]. These approaches include not only investigations that are centered on community needs and values but also the design of community-engaged recruitment strategies [53] and culturally-responsive interview guides [22]. Further testing of existing models is needed to evaluate efficacy of racially and culturally sensitive conversation guides that build connection, promote patient control, and consider religious faith and family values [29]; interpersonal approaches that explicitly invite and address patients’ experiences of racism while enhancing the quality of clinician-patient relationship [32]; and faith and social community engagement to improve advance care planning and EOL care communication in ways that are contextually sensitive and relevant [30]. 

There are several clinical implications relevant to clinicians and larger health teams, institutions, and systems. It is imperative to note that aspects of the identified themes relate to the holistic palliative care for all patients at EOL, in particular the need for person-centeredness, authenticity, cultural sensitivity, responsible and tailored pain management, building trust and connection, and understanding potential communication challenges that may arise in the context of cross-cultural differences. Clinicians must continue to develop knowledge and measurable skills in cultural humility [54], which includes a commitment to lifelong learning and self-reflexivity, mitigation of clinician–patient/community power imbalances, and institutional accountability [55,56]. Cultural humility allows intersectional identities to be honored through openness, demonstrable empathy and compassion, and an ethic of being oriented to the needs and individuality of the other person [57]. Thus, cultural humility supports clinicians to develop introspective and flexible practices to ensure person-centeredness that transcends racial and cultural differences. Safe spaces for teams to debrief and foster reflective practice about cultural challenges in care provision, the application of cultural humility, and approaches to forging an antiracist clinical culture (e.g., through supportive clinical supervision and collaboration) are imperative for high-quality palliative care delivery throughout serious illness and especially in high-stakes scenarios at EOL [25,58,59]. Furthermore, clinicians should cultivate and adopt practices of intentional stillness and solidarity with recipients of inequitable care to integrate ethical practices that mitigate ineffective responses of both inaction and delay (i.e., the opposite of antiracist practice) [60]. Our findings provide both areas of concern for clinicians and their priorities that can be considered for training and professional development purposes to support antiracist communication. 

### Strengths and Limitations

The study sample majority was White; however, a range of interdisciplinary clinicians from different racial groups was represented, suggesting that our data reflect somewhat diverse perspectives. In fact, our sample may be reflective of the limited racial and ethnic diversity of the broader United States health workforce [61]. An additional strength of the study was its success in leveraging input from diverse interdisciplinary clinicians, including chaplaincy, which are often less studied and included in such research. However, we did not draw comparisons between responses from different types of clinicians included in the study. Future research may consider exploring differences in perceptions of communication priorities with patients from minoritized groups across disciplinary lenses. Although open-text qualitative options allowed participants to respond openly without word and character count limits, the structured approach was close-ended and permitted only a focused analysis. Semi-structured approaches or in-depth interviews may have encouraged more discourse and likely uncovered additional implications. The case focused on a person with multiple minoritized identities—including being Black, Native American, a woman, and an older adult—who is facing the EOL. While we asked participants specifically about racism, we did not address issues related to ethnic or cultural stigma, ageism, or genderism. An intersectional approach to the case would likely have provided additional insights to improve antiracist research and practice. Given the sensitivity of the research topic (e.g., discrimination, racism), social desirability bias may have factored into participant responses. Finally, we focused this case on the experience of a singular patient and did not include details regarding family, caregivers, or faith or other social support communities, omitting one critical aspect of quality palliative care. We cannot generalize beyond this sample. 

## 5. Conclusions

Racism pervades every aspect of health and social care, including palliative and EOL care, increasing the suffering of racially and culturally minoritized people. Effective interpersonal communication is a backbone of clinical care—driving decision-making, intervention delivery, serious illness conversations, patient/family–clinician relationship quality, and clinical and quality of life outcomes. This study sought to elicit interdisciplinary knowledge and priorities related to communication for a patient with cancer, identities as a Black and American Indian person, and a pertinent history of racism and mistreatment in the health system. Findings support the need to equip clinicians with the sensitivity, personal awareness, and skills needed to effectively discuss patients’ racism, dismantle interpersonal and structural barriers to equitable care, and promote antiracist culture through evidence-based communication that is both empathic and person-centered. 

## Figures and Tables

**Table 1:** Case Study and Survey Questions.

Case Study		Survey Questions
Diane was recently diagnosed with end-stage ovarian cancer at the age of 71. Diane’s chart shows that she identifies as both Black and Native American. Diane had not seen a primary care provider for years because she “long ago lost trust in health care”. She recently shared with her night nurse about how she has experienced racism throughout her life—how physicians would “call her a liar” about being sick when she was a child or refuse to care for her because of her skin color. Although she shares that her abdominal pain is now severe and constant, she is quite stoic and refuses to accept pain medication from the staff. She worries that she will lose her ability “to think clearly and take care of herself if she uses that stuff”. Diane also shares that her biggest fear is dying in pain.		Understanding an individual’s history and their social determinants of health are clearly important to building trust and delivering person-centered care. How would you engage Diane in a conversation about her experiences as a Black and Native American woman who has experienced racism in the health care system? ▪ What questions would you ask? ▪ How would you build a connection? What are the challenges for you or your colleagues in discussing racism with a patient from a different race if any? Please feel free to disclose how you identify your race if you feel it is relevant to your response. What are the specific things you would say and do to regain and nurture Diane’s trust in the health care team now caring for her?

**Table 2:** Participant Demographic Characteristics.

Characteristic	*N* (%)
Sex	
Male	21(13.8)
Female	131 (86.2)
Ethnicity	
Hispanic	13 (8.6)
Non-Hispanic	139 (91.4)
Race	
American Indian/Alaskan Native	0 (0)
Asian	17 (11.2)
Black or African American	23 (15.1)
More than one Race	10 (6.6)
Native Hawaiian or Pacific Islander	3 (2.0)
White	99 (65.1)
Professional Discipline	
Chaplaincy	25 (16.4)
Nursing	73 (48)
Social Work	54 (35.6)

**Table 3:** Themes, Thematic Properties, and Illustrative Quotes.

Theme	Thematic Property	Illustrative Quotes
Person-centered, authentic, and culturally-sensitive care	Elicit the patient’s story	I would ask for permission to see if she would be agreeable to sharing her experience. (P006) I would listen to her stories of how she has been treated and managed to deal with these events throughout her life (P005)
	Validate the patient and their experiences	Acknowledge her experiences, validate her reactions, express openness to feedback about how she feels about care from our team. Make a genuine statement about our intent to truly have her values, priorities, and wishes as our guiding lights (P024) Validate patient’s experience and appreciate her trusting me in sharing her experience. Ask how we can best support and care for her during this time (P109)
	Provide authentic care	Say, “I want to help. There are ways to treat your pain, we can start slowly and try to minimize side effects. Our team will work with you to give you the best care possible.” (P017) Make a list of things that are important to Diane. Put them on the board in her room. (P011)
2. Pain control	Elicit and assess patient perspectives about pain medication and control	I would ask her to tell me more about her fear of pain medicines and pain itself (P024) Say, “Please tell me what you think about the pain medication? What are your fears and experiences?” (P021) Say, “How can I help you manage your pain?” (P23)
	Educate and provide information/resources	I would then educate her on how the use of pain medications when used correctly can help her feel better so she can continue to take care of herself (P065) Help educate on how we can help her not dying in pain, while also working with the care team to ensure she is able to think as clearly as possible (P109)
3. Approaches to build trust and connection	Offer assurance to demonstrate team’s commitment to care	What can I do to assure my availability to you in what you’re experiencing now? (P077) Assure her that we can help her be comfortable, but she will also have to trust me as a partner in her care (P080)
	Invite the patient’s perspective by asking questions about needs, preferences, and experiences	I would ask clarifying questions during visits to make sure I am hearing her concerns and statements correctly. (P030) I would ask her what I can do to support her in getting the care she needs as I would like to assist in mitigating her fears and concerns in the best way I can (P030) Say, “I can’t imagine how difficult this must be for you. How have you dealt with this in the past?” (P059) What are some things we can do to work together to provide you with quality care? What is most important to you at this time, and how can we best accommodate your needs as a Black and Native American woman? (P027)
	Convey safety, compassion, and empathy	I imagine Diane would have questions related to how her care might be different given her mistrust of the healthcare system, and I would allow time for questions and openly and honestly answer these questions showing compassion and empathy. (P006) Affirming her identity as the expert in her own life. Leaving space for sharing, sharing of experience. (P046) I would build connection by creating a “safe space” of empathy and respect for her to explore those feelings from the past and developing a plan for the future so she can care for herself. (P063)
4. Understanding communication challenges related to racial differences	Judgments and biases by clinician and/or patient	Some challenges we face with racism can be how patients feel like the doctors already formed an opinion about them without getting to know them. Patients feel unheard and that can cause more anxiety and fear in them. (P068) I find it difficult to discuss if the patient is a different race than me and is extremely upset and distrusting without allowing me time to get to know them. (P085) Because I am a person of color, I can relate to distrust toward some healthcare team members who allow their biases to determine the level of care they provide to patients (P091)
	Distrust and racism in patients	Including her in decision making and facilitating communication with the larger team. Asking her directly for explicit suggestions for what would make her feel at ease. (P149) Say, “I understand that the medical system has not treated you well in the past, but I am hoping that we can change that. Although you have no reason to trust us, I would like for you to try to give us a chance so that we can help you achieve your goals.” (P098)
	Impact of limited clinician training on patient care	Learning and educating people of different ethnicities and cultures is essential. When we isolate ourselves from differences, we lose sight of the gift of distinction. We lack understanding and become stagnant and live in silos unaware of the vast and changing world. We miss opportunities to learn new ways of doing things and may even lose opportunities to grow and use our gifts to be agents of change. (P093) I’ve experienced lack of cultural knowledge in particular with end-of-life decisions and cultural beliefs due to lack of cultural competency. (P086) I feel that our team might not have a ton of experience working with other ethnicities and cultures, so extra training would be helpful on how to be more open on asking these questions to our patients (P078) Not having diversity around them in their own lives and being uncomfortable to address the situation because they are anxious about their therapeutic communication in a “socially negative” topic that they have not experienced. (P039)
	Patient and clinician identity non-concordance	I work in a multi-racial and multi-generational environment. Some patients complain that they do not understand their nurse or nurse assistant because of the heavy accent. Some patients go as far as saying, “I want someone who can speak English.” (P105) As a White person, sometimes BIPOC do not feel comfortable disclosing they have felt discriminated against based on their race. (P109) My Caucasian colleagues find it more challenging, as being a racial minority is not an experience they have. (P039) White colleagues can’t understand it as a problem if they don’t have experience with racism (P101)
	Saying something unintentionally insensitive or offending the patient	Racial issues are always challenging because I fear insulting someone unknowingly and negatively affecting the relationship of trust. (P005) I think the challenge is perhaps the fear of saying something wrong. The intent may be therapeutic, but the execution may not be the intended outcome. (P052)

## Data Availability

The data presented in this study are available on request from the corresponding author. The data are not publicly available due to ongoing analysis.
